# DeepAProt: Deep learning based abiotic stress protein sequence classification and identification tool in cereals

**DOI:** 10.3389/fpls.2022.1008756

**Published:** 2023-01-12

**Authors:** Bulbul Ahmed, Md Ashraful Haque, Mir Asif Iquebal, Sarika Jaiswal, U. B. Angadi, Dinesh Kumar, Anil Rai

**Affiliations:** ^1^ Division of Agricultural Bioinformatics, ICAR-Indian Agricultural Statistics Research Institute, New Delhi, India; ^2^ Division of Computer Application, ICAR-Indian Agricultural Statistics Research Institute, New Delhi, India; ^3^ Department of Biotechnology, School of Interdisciplinary and Applied Sciences, Central University of Haryana, Mahendergarh, Haryana, India

**Keywords:** abiotic stress, activation function, deep learning, web-server, mobile application

## Abstract

The impact of climate change has been alarming for the crop growth. The extreme weather conditions can stress the crops and reduce the yield of major crops belonging to Poaceae family too, that sustains 50% of the world’s food calorie and 20% of protein intake. Computational approaches, such as artificial intelligence-based techniques have become the forefront of prediction-based data interpretation and plant stress responses. In this study, we proposed a novel activation function, namely, Gaussian Error Linear Unit with Sigmoid (SIELU) which was implemented in the development of a Deep Learning (DL) model along with other hyper parameters for classification of unknown abiotic stress protein sequences from crops of Poaceae family. To develop this models, data pertaining to four different abiotic stress (namely, cold, drought, heat and salinity) responsive proteins of the crops belonging to poaceae family were retrieved from public domain. It was observed that efficiency of the DL models with our proposed novel SIELU activation function outperformed the models as compared to GeLU activation function, SVM and RF with 95.11%, 80.78%, 94.97%, and 81.69% accuracy for cold, drought, heat and salinity, respectively. Also, a web-based tool, named DeepAProt (http://login1.cabgrid.res.in:5500/) was developed using flask API, along with its mobile app. This server/App will provide researchers a convenient tool, which is rapid and economical in identification of proteins for abiotic stress management in crops Poaceae family, in endeavour of higher production for food security and combating hunger, ensuring UN SDG goal 2.0.

## 1 Introduction

The drastic climatic changes due to global warming after the 1980s lead to significant yield loss in various crops (Lobell et al., 2011). The Poaceae family of crops, especially rice, wheat, and maize, which account for ~50% of the world’s food calories and 20% of its protein intake (Erenstein et al., 2022), are highly susceptible to abiotic stress like heat, salinity, drought, and cold (Landi et al., 2017). On the other hand, due increasing global population, which may be around 9.5 billion by 2050, the current food availability gap requires a dramatic increase in food by 2050 (Cobb et al., 2013). It is already well known that environmental stressors negatively regulate the growth and development of plants leading to substantial yield and quality losses (Boyer, 1982; Palanog et al., 2014, Gupta et al., 2021). A recent study suggests that climate change could reduce global crop yields by 3–12% by mid-century, and by 11–25% by the century’s end, under a vigorous warming scenario (Sue Wing et al., 2021).

Stresses in plants, like drought, salinity, cold, etc. are their defensive states which result from deviations from their optimal growth conditions (Jansen and Potters, 2017). These stresses lead to a loss in yield, thus affecting food security, especially in the current scenario of climate change (Rico-Chávez et al., 2022). Therefore, there is a need to conceive comprehensive strategies for trait improvement of important crops, especially of the Poaceae family, under adverse climatic conditions. Artificial intelligence (AI)- based machine learning techniques have become the forefront of prediction-based data interpretation and plant stress responses (Gill et al., 2022). Analyses of high-throughput genomic data in recent years, like, genes, transcripts, proteins, metabolites, etc., require advanced analytical methods for proper associations and interactions. The promising computational power in terms of artificial intelligence (AI) based methodologies had been a promising means for analyzing various plant stress mechanisms (Fenu and Malloci, 2021). Also, machine learning (ML) based methodologies for identifying DNA N6-methyladenine sites of plant genomes (Hasan et al., 2021), a deep-learning-based hybrid framework for identifying human RNA N5-methylcytosine sites (Hasan et al., 2022), solving classification problems in molecular data like amino acid sequence, protein sequences and structures (Cai et al., 2020; Xu et al., 2020; Gelman et al., 2021; Sridevi and Kanimozhi, 2021; Wang, 2022; Ding et al., 2022) proves the versatility of ML methodologies. The use of ML-based studies to identify, classify, and predict various stresses in plants are well reported, *namely*, in basil, coriander, parsley, baby-leaf, coffee, pea, and maize for water stress (Niu et al., 2021; Zahid et al., 2022), in *Arabidopsis thaliana* for heat, cold, salt, and drought (Kang et al., 2018), salt stress in rice (Das et al., 2020) and wheat (Moghimi et al., 2018), drought stress in *Bromus inermis* (Dao et al., 2021), and biotic stresses in soybean (Venal et al., 2019), etc.

Various studies have been done using ML/Deep Learning techniques to classify stress-responsive varieties in corn using deep convolutional neural networks (Ghosal et al., 2018; Khaki et al., 2019), neural networks (Etminan et al., 2019), linear mixed model (Chen et al, 2012) and CNN (An et al., 2019), etc. However, there are limited resources of deep-learning-based prediction models for the abiotic stress protein sequence of the Poaceae crop family. Therefore, we developed a deep learning approach for the classification of the abiotic stress protein sequence of this family. In addition, we developed a novel activation function, namely, *sielu* that has increased accuracy as compared to the existing models. The same has been applied to the stress datasets. Most of the data under study were benchmark data collected from Uniprot. Although, the DL model works well in the structure, unstructured, and complex features of the dataset, however, it requires a large dataset to train the model (Elaraby and Elmogy, 2016). It also uses different optimization techniques, weight functions, loss functions, and activation functions during model development (Wen et al., 2018; Salman and Liu, 2019). During model building, an activation function plays an important role in boosting the performance of the model as this helps in the activation or deactivation of neurons (Benvenuto and Piazza, 1992; Sarker, 2021). DL model without an activation function converges to linear regression model. Several activation functions like sigmoid, ReLU, LeakyReLU, Tanh, and Softmax have been reported in the literature (Xu et al., 2015; Hendrycks and Gimpel, 2016; Agarap, 2018; Pratiwi et al., 2020) are being used in building DL for the classification and prediction (Li et al., 2018; Armenteros et al., 2019; Bileschi et al., 2022). Some of the major limitations of these activation functions are the vanishing gradient, loss of neurons, and problems in training small datasets (Srinivasan et al., 2019).

In this study, we proposed a novel activation function, named Gaussian Error Linear Unit with Sigmoid *(SIELU)* to overcome issues related to the activation function. Further, we have built a DL model using the proposed activation function for the prediction of abiotic stresses, i.e. heat, drought, cold, and salinity responsive protein sequences from the crops of the Poaceae family. Also, a Web server has been developed, which can be extensively used by researchers/breeders for the development of abiotic stress resistance varieties of the crops of the Poaceae family for increasing agricultural production and productivity. In the future, there is a scope for developing different weight initialization techniques, activation functions, optimizers, etc. for more efficient classification using deep learning models.

## 2 Materials and methodology

### 2.1 Activation function

A series of studies have been carried out related to various activation functions and their performance in DL network building. The extensively used activation functions in DL models are *Sigmoid*, *Tanh*, *ReLU*, *LeakyReLU, SoftMax*, etc. (Dunn et al., 2011).

*Sigmoid function*: For any given input of data, the sigmoid maps to 0 or 1. If a given input goes above the predetermined threshold value, it will give output as 1, otherwise, 0, *i.e.*, the neuron will remain deactivated. Scientifically, it has been proven that the human brain functions like the sigmoid function for differentiating and classifying objects (Pratiwi et al., 2020). Mathematically, it is expressed as:


$$f\left(x\right)=\frac{1}{1+e^{-x}}$$

*Tanh function*: It is similar to the Sigmoid function with little modification for the output and expressed mathematically as (LeCun et al., 2012):


$$f\left(x\right)=\frac{2}{1+e^{-2x}}-1$$

*Rectified Linear Unit (ReLU):* This activation function uses stochastic gradient descent for back-propagation by adjusting the learning rate and minimizing the errors during training a model. Also, it provides a better solution without decaying the hidden layers by adjusting the learning rate and minimizing the error differentiation by removing all the negative values in back-propagation. Mathematically, *ReLU* can be expressed as (Agarap, 2018):


$$f\left(x\right)=\{\begin{array}{c} x,\;\;\;for\;x\geq 0\\ 0,\;for\;x<0 \end{array}$$

*Leaky Rectified Linear Unit (LaekyReLU):* It is an extension of *ReLU i.e.*, by using some value, say *σ*=0.01 that makes the neuron active instead of deactivating for zero values. Mathematically, the *LeakyReLU* function is expressed as (Xu et al., 2015):


$$f\left(x\right)=\{\begin{array}{c} x,\;\;\;\;\;\;\;\;\;\;\;\;\;\;\;for\;x\geq 0\\ \sigma *x,\;\;for\;x<0 \end{array}$$

*Softmax function:* It gives the probability of each true class and is expressed as (Kanai et al., 2018):


$$f\left(x_{j}\right)=\frac{e^{x_{j}}}{\sum_{k=1}^{k}e^{x_{k}}}$$

Many other activation functions have been developed which are mainly derived from the above activation functions such as Gaussian Error Linear Unit (*gelu*) (Hendrycks and Gimpel, 2016), a multi-layer perceptron model with a *sigmoid, tanh, conic section, and radial bases function (RBF)*, etc. (Karlik and Olgac, 2011; Cai et al., 2015).

### 2.2 Proposed Gaussian error linear unit with sigmoid activation function (SIELU) activation function

It may be noted that the *Tanh* activation is used in the Cumulative Distribution Function of GELU. Also, *Tanh* activation function is reported to perform better than sigmoid (Szandała, 2021; Ingole and Patil, 2020; Jiang et al., 2020) but takes more time. However, in the prediction of high-dimension datasets, computational time is one of the crucial factors. It has been pointed out that the sigmoid function requires less time and is computationally inexpensive by approximating its polynomial for positive outputs (Wang et al., 2020). Further, the sigmoid function is computationally easy to perform. Therefore, a thorough investigation was done to derive a novel activation function *i.e.*, *SIELU* from the *GELU* function.

An approximation of normal distribution (q) was carried out in 1955 for the first time by (Hastings, 1955; Brophy, 1985) which was expressed as:


$$q=\frac{1}{\sqrt{2\pi }}\int_{-\infty }^{\infty }e^{-\frac{1}{2}t^{2}}\partial t;0\leq q\leq 0.5$$

Hence $X^{*}\left(q\right)=\eta -\left\{\frac{\alpha_{0}+\alpha_{1}\eta }{1+b_{1}\eta +b_{2}\eta^{2}}\right\};\;\eta =\sqrt{ln\frac{1}{q^{2}}}$ ;

where , *α*_0_=2.30753 ,  *α*_1_=0.27061 ,  *b*_1_=0.99229 ,  *b*_2_=0.04481 or $\;\;\;\;\;\;\;X^{*}\left(q\right)=\eta -\left\{\frac{\alpha_{0}+\alpha_{1}\eta +\alpha_{2}\eta^{2}}{1+b_{1}\eta +b_{2}\eta^{2}+b_{3}\eta^{3}}\right\}$ ,

were, *q*→ *normal* *distribution* ,  *t*→ *time*,  *α*_0_=2.515517 ,  *α*_1_=0.802853 ,  *α*_1_=0.010328 ,  *b*_1_=1.432788 ,  *b*_2_=0.189269 , *b*_2_=0.001308 (Hastings., 1955).

With the advancement of technology, a more accurate approximation was introduced by estimating the standard normal deviated distribution *z*  by (Zelen and Severo, 1964) followed by Emerson, 1979.


$$z=t-\left\{\frac{C_{0}+C_{1}t+C_{2}t^{2}}{1+d_{1}t+d_{2}t^{2}+d_{3}t^{3}}\right\}+e\left(p\right)$$

where, $\;t=\sqrt{ln\frac{1}{p^{2}}\;}$ and  |*e*(*p*)|<4,5×10^−4^ ,  *C*_0_=2.515517 ,  *C*_1_=0.802853 ,  *C*_2_=0.010328 ,  *d*_1_=1.43288 ,  *d*_2_=0.189269 ,  *d*_3_=0.001308 .

Later, in 2008, standard normal deviated distribution to approximate the function was given by Kiani and co-workers (Kiani et al., 2008) as follows:

$\text{Φ}\left(x\right)=\frac{1}{2}\left\{1-erf\left(\frac{-z}{\sqrt{2}}\right)\right\}$ ; −*∞*<*z*<*∞* where $erf\left(z\right)=\int_{0}^{z}\frac{2}{\sqrt{\pi }}e^{-t^{2}}\partial t$ ; −*∞*<*z*<*∞* .

Moreover, the approximation of Φ(*x*)−0.5 with absolute error<  3×10^−5^ (Bagby, 1995) is estimated from:


$$\text{Φ}\left(x\right)-0.5\approx 0.5\left(1-\frac{1}{30}\right){\left[7\times \exp\left(\frac{-z^{2}}{2}\right)+16\times exp\left\{-z^{2}\left(2-\sqrt{2}\right)\right\}+\left(7+\frac{\pi z^{2}}{4}\right)\times exp\left(-z^{2}\right)\right]}^{0.5}$$

Our proposed Gaussian Error Linear Unit with Sigmoid (*SiELU*) was constructed by modifying the GELU function as follows:


(1) $$\text{GELU}:\text{f}\left(x\right)=0.5x\left[1+tanh\left\{\sqrt{\frac{2}{\pi }}\times \left(x+0.044715x^{3}\right)\right\}\right]$$

Let $tanh\left\{\sqrt{\frac{2}{\pi }}\times \left(x+0.044715x^{3}\right)\right\}=\text{tanh}\left(y\right)$ where, $y=\sqrt{\frac{2}{\pi }}\times \left(x+0.044715x^{3}\right)$ On simplification of the equation (1):


f(x)=0.5x[1+tanhy]

Tanh and Sigmoid functions are mathematically defined as:


(2) $$Tanh\left(x\right)=\frac{e^{x}-e^{-x}}{e^{x}+e^{-x}}$$

(3) $$Sigmoid\left(x\right)=\frac{1}{1+e^{-x}}$$

On further simplification of the equation (2),


(4) $$Tanh\left(x\right)=\frac{e^{x}-e^{-x}+\;e^{-x}-e^{-x}}{e^{x}+e^{-x}}=1-\frac{2e^{-x}}{e^{x}+e^{-x}}$$

By dividing numerator and denominator by e^-x^, equation (4) is changes to:


(5) $$Tanh\left(x\right)=\frac{e^{x}-e^{-x}+\;e^{-x}-e^{-x}}{e^{x}+e^{-x}}=1-\frac{2}{e^{2x}+1}=1-2\times Sigmoid\left(-2x\right)$$

From equation (1) , f(*x*)=0.5*x*[1+*tanhy*] Now, equating sigmoid with *tanh* function and simplifying, we get:


$$sigmoid\left(y\right)=\frac{\tanh\left(\frac{y}{2}\right)+1}{2}-1$$

2×sigmoid(2y)−1=tanh(y)

Finally, the *SiELU* can be expressed as:


$$SiELU\text{f}\left(x\right)=0.5x\left[1+2\times sigmoid\left\{2\times \sqrt{\frac{2}{\pi }}\left(x+0.044715x^{3}\right)-1\right\}\right]$$

On simplification, we got the Gaussian Error Linear Unit with Sigmoid activation function, termed *SIELU* as follows:


$$SiELU\text{f}\left(\text{x}\right)=0.5x\left[2\times sigmoid\left\{2\times \sqrt{\frac{2}{\pi }}\left(x+0.044715x^{3}\right)\right\}\right]$$

### 2.3 Deep learning model with proposed activation function

#### 2.3.1 Data collection and pre-processing:

Abiotic stress responsive protein sequence data, *namely*, “salt stress”, “drought stress”, “heat stress” and “cold stress” of the *Poaceae* family were retrieved using Boolean operator from the public domain (Uniprot database: https://www.uniprot.org/). Also, the negative dataset of the corresponding stress conditions has been downloaded with the NOT operator. A total of 46 features were extracted from each of these sequences using the bio-python package, (Cock et al., 2009) (**Table 1**). All the redundant sequences were removed with a similarity of 80% or more using the CD-Hit suite (Huang et al., 2010). For pre-processing the dataset, StandardScaler was used to transform these datasets into Standard Normal Distribution (SND) of the data having zero mean and unit variance, which reduces the biases of the models (Ahsan et al., 2021; Karlaš et al., 2022; Cha and Bae, 2022).

**Table 1:** Set of features under study.

Sl. No.	Features	Sl. No.	Features
1	Composition of Alanine (A)	24	C*- Nitrosylation (Nito C)*
2	Composition of Arginine (R)	25	Total N*itrosylation (Total Nitro)*
3	Composition of Asparagine (N)	26	A- *Nitrotyrosine (YNO A)*
4	Composition of Aspartate (D)	27	B- *Nitrotyrosine (YNO B)*
5	Composition of Cysteine (C)	28	C- *Nitrotyrosine (YNO C)*
6	Composition of Glutamine (Q)	29	Total *Nitrotyrosine* (YNO Total)
7	Composition of Glutamate (E)	30	SUMOylation I (SUMO I)
8	Composition of Glycine (G)	31	SUMOylation II (SUMO II)
9	Composition of Histidine (H)	32	SUMOylation III (SUMO III)
10	Composition of Isoleucine (I)	33	Total SUMOylation (SUMO Total)
11	Composition of Leucine (L)	34	Amino acid number
12	Composition of Lysine (K)	35	Number of negative amino acids
13	Composition of Methionine (M)	36	Number of positive amino acids
14	Composition of Phenylalanine (F)	37	Molecular weight
15	Composition of Proline (P)	38	Theoretical PI
16	Composition of Threonine (T)	39	Number of carbon atoms
17	Composition of Serine (S)	40	Number of hydrogen atoms
18	Composition of Tryptophan (W)	41	Number of nitrogen atoms
19	Composition of Tyrosine (Y)	42	Number of oxygen atoms
20	Composition of Valine (V)	43	Number of sulphur atoms
21	Coiled-coil domain (CCD)	44	Instability index
22	A*- Nitrosylation (Nito A)*	45	Aliphatic index
23	B*- Nitrosylation (Nito B)*	46	Grand average hydropathy (GRAVY)

This data pertains to various features that were scaled down and standardized as follows to achieve consistency in the varying range of datasets:


$$Scaling\left(\hat{x}\right)=\frac{x-\text{min}\left(x\right)}{\max\left(x\right)-\text{min}\left(x\right)}$$

$$\text{Standardization}\left(Z\right)=\frac{x-\mu }{\sigma };\;\mu =0;\;\sigma^{2}=1\;$$

where, *Z* is standard normalization with *x*  variables, *μ* mean, and *σ* ^2^  variance (Tauber and Sánchez, 2002).

**Figure 1:**
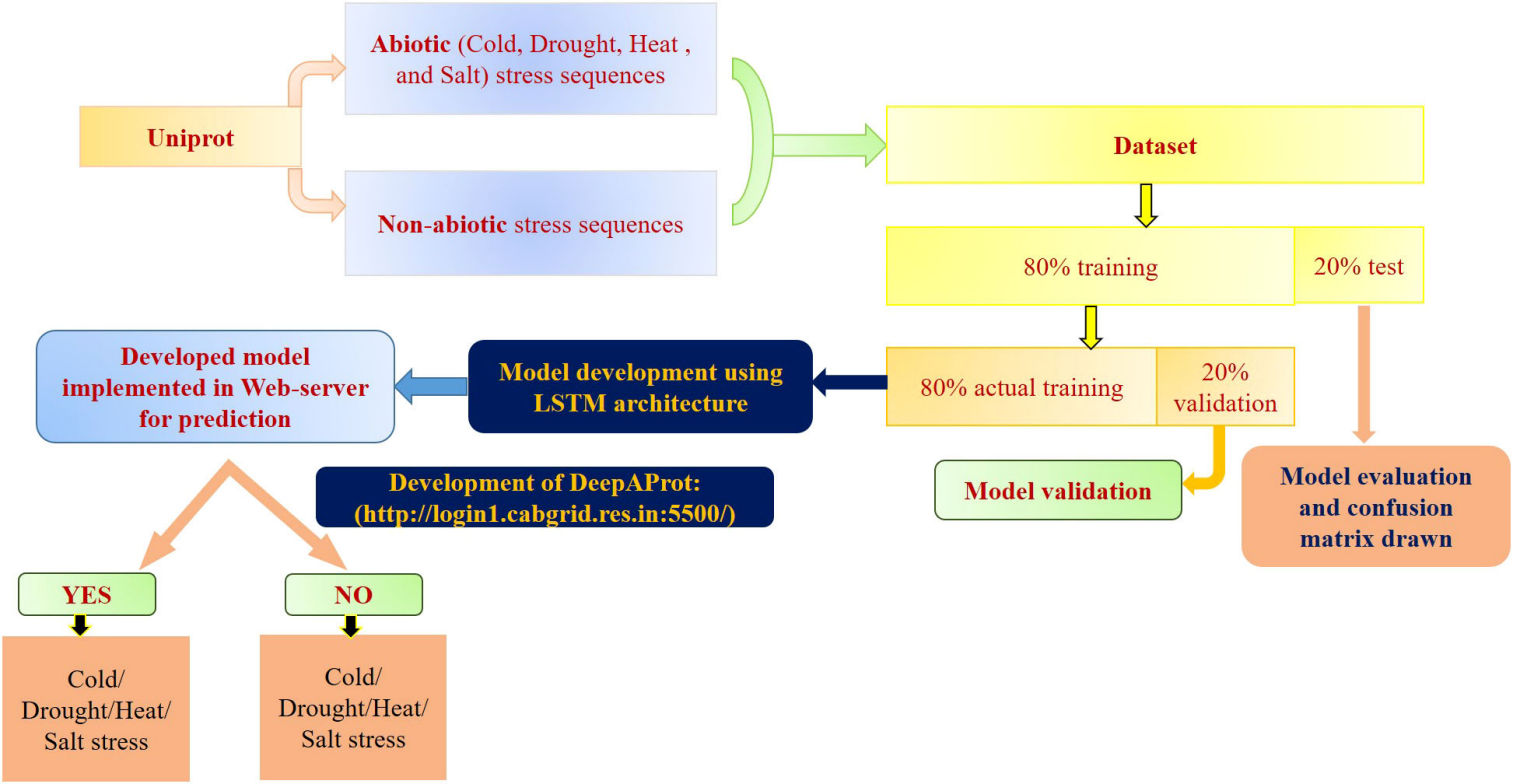
Schematic workflow for model implementation in the development of DeepAProt.

For different layers and epochs, first, stratified sampling was performed, followed by random selection of the training dataset using python script, sklearn library. Different combinations of training:test sets, like, 70:30, 80:20, and 90:10 were made, and finally we proceeded with 80:20 based on the accuracy parameter (Gholamy et al., 2018; Akarsh et al., 2019; Pham et al., 2020; Nguyen et al., 2021; Gu et al., 2022). From this training data, actual training data and drop-out prediction data were retained at 80:20. Fine tuning of weight initializer, layers, epochs, and activation function was carried out in the model to assess the model performance in each epoch. For the given datasets of four stresses, different machine learning algorithms such as SVM, RF, LSTM models were applied using GeLU. For SVM models, polynomial kernel function, 0.01 coeff, and 5-fold StratifiedKFold were used in SVM models for maximum efficiency. In the case of Random Forest, we used a minimum of 0.1 leaf weight with 5-fold StratifiedKFold. For the deep learning model, 150 units, *He* normal kernel initializers, *gelu* activation function, and the proposed activation function *i.e*., *sielu* were used for comparative analysis in input layers. In the case of the hidden layer, 50 units, 0.02 dropout, and sigmoid activation with 1 unit for binary classification (in the output layer) were employed. During the model compilation, an Adam optimizer and mean square error loss function were used with 500 epochs. The schematic diagram of the methodology is represented in **Figure 1**.

### 2.4 Model evaluation indicators

For model evaluation, measures such as accuracy, precision, recall, F1 Score, specificity, and MCC were applied. These parameters were calculated for all four abiotic stresses for SVM, RF, LSTM with GeLU, and LSTM with SieLU activation functions. These are expressed as follows:


$$Sensitivity=\left(\frac{TP}{TP+FN}\right)\;\times \;100$$

$$Precision=\left(\frac{TP}{TP+FP}\right)\;\times \;100$$

$$F_{1}=\;2\;\times \left(\frac{Precision\;\times \;Recall}{Precision+Recall}\right)$$

$$Recall=\left(\frac{TP}{TP+FN}\right)$$

$$Accuracy=\;\left(\frac{TP+TN}{TP+\;TN+FP+FN}\right)\;\times \;100$$

$$MCC=\left(\frac{TP\;\times \;TN-FP\;\times \;FN}{\sqrt{(TP}+FP)\left(TP+FN\right)\left(TN+FP\right)\left(TN+FN\right)}\right)\;\times \;100$$

where, TP = True Positive, TN = True Negative, FP = False Positive, FN = False Negative.

## 3 Results and discussion

A thorough screening of “salt stress”, “drought stress”, “heat stress” and “cold stress” associated protein sequences from the Poaceae family retrieved from the public domain resulted in a total of 739 positive and 1305 negative protein sequences of cold stress, 642 positive and 1284 negative protein sequences of drought stress, 977 positive and 1305 negative protein sequences of drought stress, and 473 positive and 946 negative protein sequences of salt stress. For these datasets, 46 protein sequence features were extracted (**Table 1**) using the bio-python package. These features were scaled down and standardized. The scaling method was used followed by the transformation of feature information into 0 to 1 to reduce the dominance of one feature over others (Beljkas et al., 2020).

The DL models were built using Sigmoid, Tanh, ReLU, LeakyReLU, SoftMax using the above data set and their performance was evaluated with respect model using the proposed SIELU activation function. Also, models were built based on these stress-associated datasets with different machine learning algorithms, namely, SVM, RF, and DL with GeLU activation function were also evaluated with the model using the proposed SEILU activation function. Off course, the proposed SIELU activation function was used in LSTM along with other fine-tuning hyper-parameters for the model development of four different abiotic stress protein sequence datasets of the Poaceae family. All these developed models were subjected to five-fold cross-validation.

The performance of these models was recorded from the test dataset in the form of a confusion matrix for calculating the various evaluation measures, *namely*, accuracy, precision, recall, F1 Score, specificity, and MCC. The following points emerged from this analysis:

It was observed that, for the cold stress dataset, accuracy and MCC were highest for LSTM with the proposed activation function, SieLU, i.e., 95.11% and 0.90, respectively for testing and 99.20% and 0.98 for the training dataset. LSTM with GeLU activation function gave an accuracy of 94.62% and MCC of 0.89 for the testing dataset and 100% accuracy and MCC of 0.89 in the training dataset. The performance of RF was lowest, i.e., 87.53% accuracy and 0.74 MCC for the testing dataset, accuracy of 88.43% and MCC of 0.75 for the training dataset (**Table 2**).

**Table 2:** Comparison of LSTM with *sielu* and *gelu*, SVM, and RF for different abiotic stress-associated protein sequences. The figures in bold denote the evaluation parameters of the best fit model for given stress.

Samples	Models	Accuracy (%)		Precision (%)		Recall (%)		F1 Score (%)		Specificity (%)		MCC	
		Training	Testing	Training	Testing	Training	Testing	Training	Testing	Training	Testing	Training	Testing
**Cold**	***LSTM (sielu)***	**99.2**	**95.11**	**98.97**	**92.95**	**98.8**	**94.16**	**98.88**	**93.55**	**99.43**	**95.69**	**0.98**	**0.9**
	LSTM (*gelu*)	100	94.62	100	93.42	100	92.21	100	92.45	100	96.08	0.89	0.89
	SVM	97.25	94.38	100	96.45	92.29	88.31	95.99	92.2	100	98.04	0.94	0.88
	RF	88.43	87.53	97.14	96.4	69.69	69.48	81.15	80.75	98.86	98.43	0.75	0.74
**Drought**	**LSTM (*sielu*) **	**97.79**	**80.78**	**97.11**	**79.31**	**95.92**	**64.79**	**96.5**	**71.32**	**98.67**	**90.12**	**0.95**	**0.58**
	LSTM (*gelu*)	100	78.18	100	71.32	100	68.31	100	69.78	100	83.95	0.53	0.53
	SVM	85.39	75.06	96.6	82.86	56.91	40.85	71.62	54.71	99.04	95.06	0.67	0.45
	RF	72.08	67.53	88.76	84	15.83	14.79	26.87	25.14	99.04	98.35	0.3	0.26
**Heat**	**LSTM (*sielu*)**	**99.12**	**94.97**	**99.1**	**95.31**	**98.85**	**92.89**	**98.97**	**94.09**	**99.33**	**96.54**	**0.98**	**0.9**
	LSTM (*gelu*)	100	93.65	100	92.42	100	92.89	100	92.66	100	94.23	0.87	0.87
	SVM	88.71	87.31	89.3	87.17	83.57	82.74	86.33	84.89	92.54	90.77	0.77	0.74
	RF	85.64	87.96	95.59	97.97	69.58	73.6	80.53	84.05	97.61	98.85	0.72	0.77
**Salt**	**LSTM (*sielu*)**	**98.06**	**81.69**	**97.28**	**72.63**	**96.76**	**72.63**	**97.01**	**72.63**	**98.69**	**86.24**	**0.96**	**0.59**
	LSTM (gelu)	100	80.63	100	73.81	100	65.26	100	69.27	100	88.36	0.55	0.55
	SVM	75.49	75.35	63.56	62.63	61.54	65.26	62.53	63.91	82.43	80.42	0.44	0.45
	RF	84.92	79.93	94.4	88	58.09	46.32	71.92	60.68	98.28	96.83	0.66	0.53

For the drought-responsive protein sequences, the performance of LSTM with SieLU activation function was best with accuracy and MCC as 80.78% and 0.58, respectively for the testing dataset and 97.79% accuracy and MCC 0.95 for the training dataset. This was followed by LSTM with GeLU activation function (Accuracy 78.18%, MCC 0.53 for testing dataset and Accuracy of 100% and MCC 0.53 for training dataset), SVM (Accuracy 75.06%, MCC 0.45 for testing and Accuracy 85.39% and MCC 0.67 for training dataset) and RF (Accuracy 67.53, MCC 0.26 for testing dataset and Accuracy 72.03% and MCC 0.30 for training dataset).

In the case of heat stress also, we found LSTM with a novel activation function, SieLU to perform best with 94.97% accuracy and 0.90 MCC for the testing dataset while an Accuracy of 99.12% and MCC 0.98 for the training dataset. The accuracies for LSTM (GeLU), SVM, and RF were 94.97%, 93.65%, and 87.31%, and 87.96% respectively for the testing dataset whereas for the training dataset, it was found as 99.12%, 100%, 88.71%, and 85.64% respectively, while MCCs were 0.90, 0.87, 0.74, and 0.77 respectively for testing dataset whereas for training it was 0.98, 0.87, 0.77, and 72 respectively. A similar trend was observed in performance for the salt stress dataset also. Accuracy of LSTM (SieLU), LSTM (GeLU), SVM, and RF were 81.69%, 80.63%, 75.35, and 79.93 respectively for the testing dataset, whereas for the training dataset, it was 98.06%, 100%, and 75.49%, and 84.92% respectively. **Table 2** delineates the performance of models in detail.

**Figure 2:**
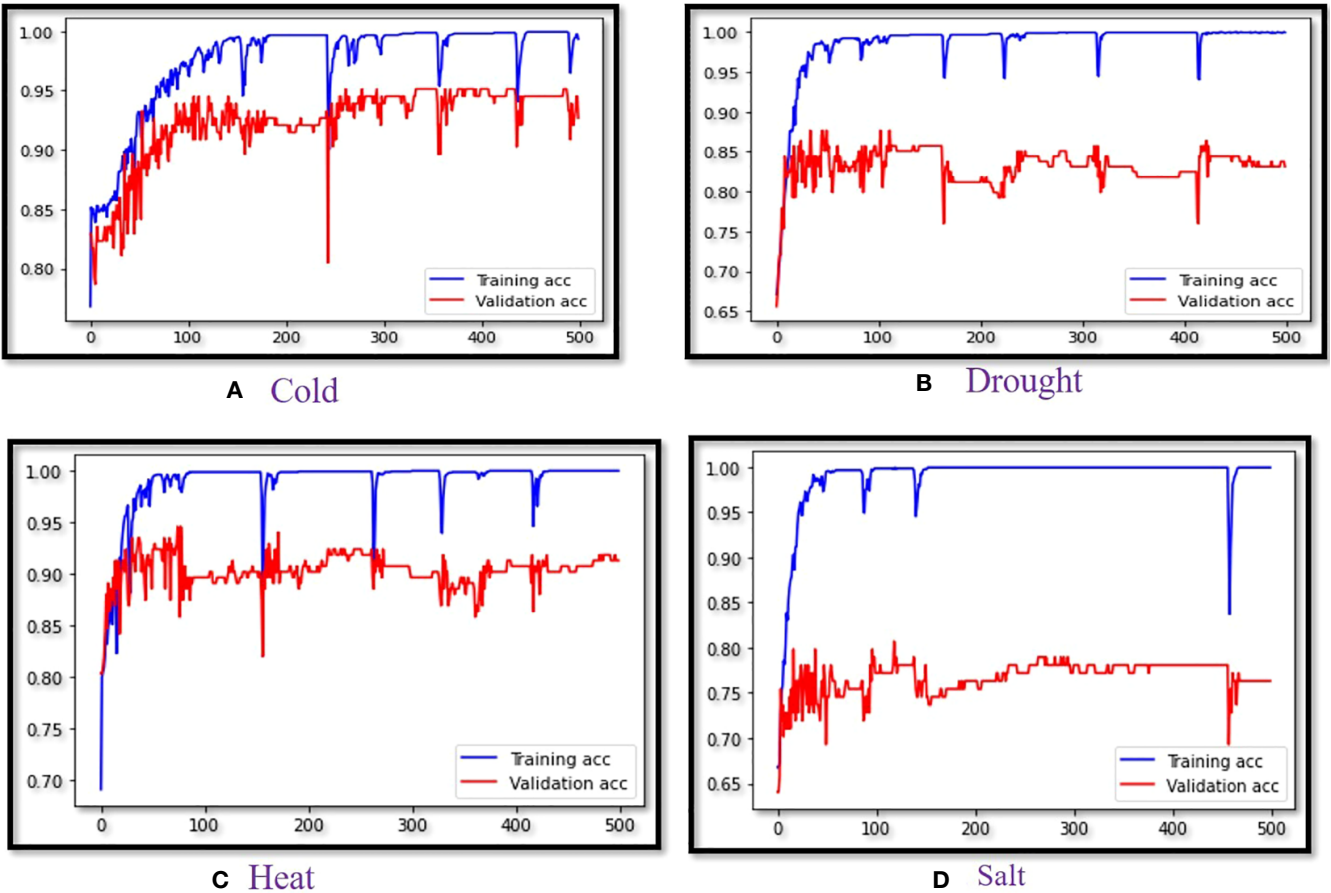
Validation curve of LSTM (SiELU) for **(A)** Cold stress , **(B)** Drought stress, **(C)** Heat stress and **(D)** Salt stress.

Training accuracy vs. validation accuracy was captured for each epoch in which performance LSTM (SieLU) was found to be superior for all four abiotic stress datasets (**Figure 2**). For the binary classification of four different abiotic datasets, we used a precision-Recall graph (**Supplementary Figure 1**) for measurement of the performance of our developed models (Flach and Kull, 2015; Boyd et al., 2012). Analogously, the ROC (Receiver Operating Characteristics) curve shows the comparison of the performance of the developed ML/DL models for all the abiotic stress datasets (**Supplementary Figure 2**) (Majnik and Bosnić, 2013). Therefore, it can be concluded that the LSTM model with the proposed *SIELU* activation function outperformed in all datasets as compared to the other competitive models used in this study for classifying protein sequences. Further, these models were also cross-validated with the benchmark heart disease dataset available in the UCI machine learning repository which consists of 303 samples with the 13 most significant features (Otoom et al., 2015). The results showed LSTM (SiELU) to have the highest accuracy (94.74%) and MCC (0.89) as compared to other machine learning models, namely, LSTM (GELU), SVM and RF which showed MCC of 0.86, 0.57 and 0.53, respectively.

### 3.1 DeepAProt: Web implementation

A web-based tool, named as *DeepAProt*, was developed using the Application Programming Interface (API) flask for the deployment of these DL models. In this web server, the best model for each of the stress-responsive datasets was implemented at the backend to develop a web server for the prediction of related stress-responsive proteins. The architecture of a web-based tool followed the standard three-tier architecture, *namely*, presentation, web-API, and application layer. The presentation layer is the user interface of the tool which was implemented using HTML and CSS languages. In web-API, a REST API was developed for deploying the model in the server. This layer was implemented using the Python programming language. Finally, the application layer contains the models for the end users, making it more user-friendly for easy use and access. For its application at remote locations, a mobile app “DeepAProt app” was also developed. “DeepAProt app” is developed using Java and XML as a front-end mobile app using android studio. For the interface of the web tool, the Python Flask framework has been used. The Back-end web tool is developed on a python framework using a deep learning module *i.e.*, TensorFlow. This app has the provision to upload protein sequence data in *fasta* format for analysis and the result will be presented in a tabular form regarding the given protein sequences association with abiotic stresses such as cold, drought, heat, and salt. In this app, a provision was also made to download and help document and sample data. It makes use of HTML (Peroni et al., 2017), javascript (Delcev and Draskovic, 2018), and CSS (Genevès et al., 2012) at the back-end and front-end to classify any protein sequence (in *fasta* format) that has to be upload as input by biologists.

**Figure 3:**
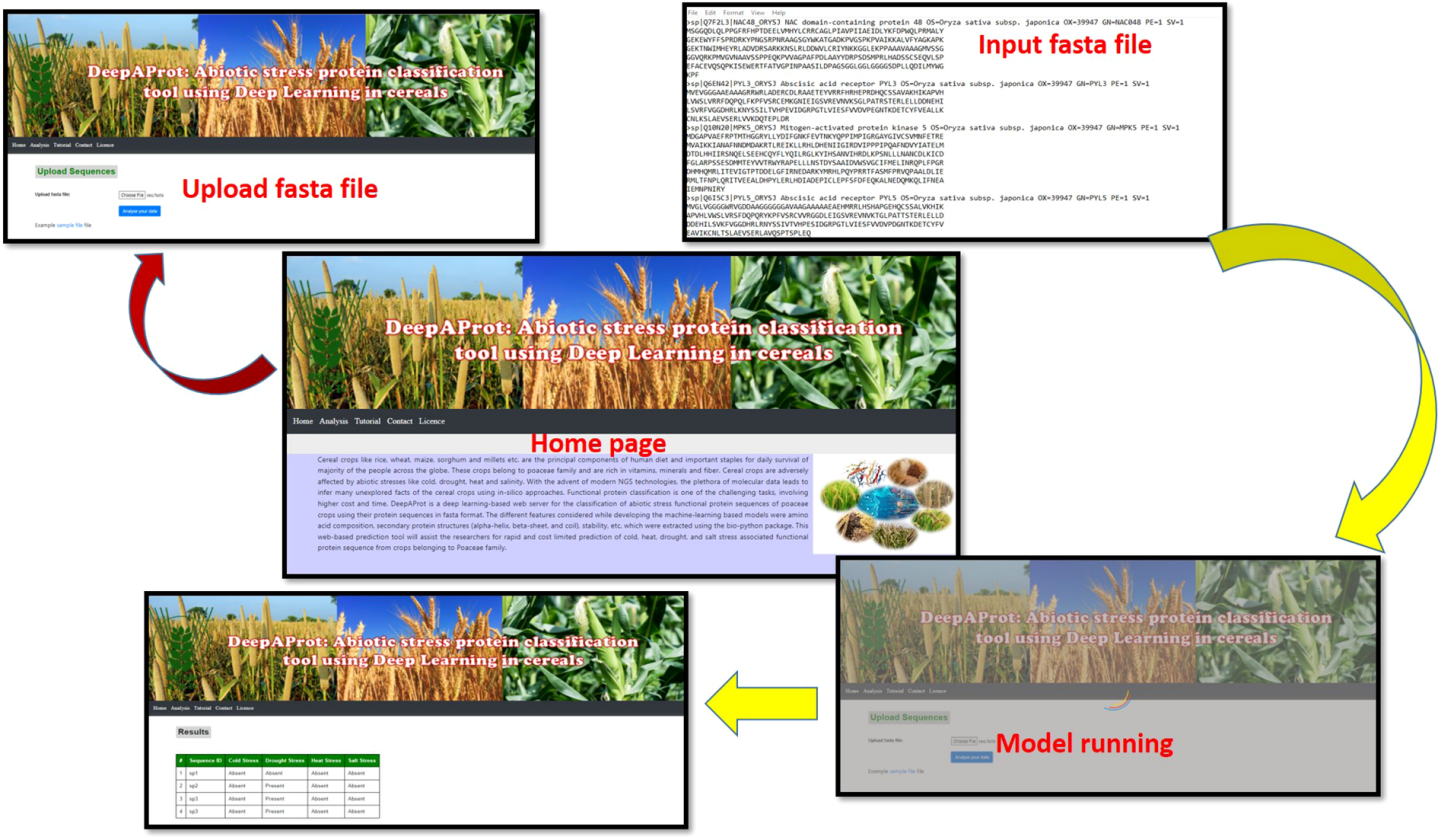
Interface for use of DeepAProt.

The user can select either of the abiotic stresses, (i.e., heat/cold/salt/drought) followed by uploading the sequence. Once the raw protein sequence is uploaded in *fasta* format, the output classifies the sequences to the predicted category. This web server is user-friendly and freely accessible at http://login1.cabgrid.res.in:5500/. **Figure 3** shows the interface of this web-implemented server and its usage. This web-based tool helps the biologist to classify the unknown protein sequence to the respective class of abiotic stress. Also, the developed mobile app can be popularized for easy and quick handling of data for the identification of stress. It can be downloaded from the Homepage.

As classification and prediction of proper abiotic stress protein sequences help the biologist to implement it in crop improvement. Machine learning and deep learning models help to find out the abiotic stress protein sequences in a cost and effective manner. However, most biologists do not have enough knowledge about machine learning and deep learning to predict the proper abiotic stress protein sequences. Therefore, our models help them to distinguish between the abiotic stress and non-abiotic stress protein sequence that comes from the sequencing laboratory directly.

## 4 Conclusion

In this study, we proposed a novel activation function name SIELU which was used to build the DL model along with other hyperparameters. The performance of this novel activation function has been studied using public domain data to predict stress-responsive proteins under four abiotic stresses, *namely*, cold, heat, salinity, and drought from the major crops of the Poaceae family. Further, a comparative analysis was carried out between *SVM, RF*, and *LSTM* with *GELU*, and *SIELU* activation functions. It has been observed that LSTM with *SIELU* activation function outperformed as compared to other competitive models used in this study. Hence, LSTM with *SIELU* models was implemented in the form of web servers for the classification of unknown protein sequences into different abiotic stresses of crops from the Poaceae family. This work can be of immense use for plant breeders for *in silico* identification of the stress-responsive proteins in crops of the Poaceae family, leading to the rapid development of abiotic stress-resistant varieties.

**Resource used:** The research was carried out using python programming packages, version 3.7.8. Also, for the graphical user interface (GUI), Anaconda Repository was used for coding these models in a Jupyter notebook with necessary python libraries. All these model buildings have been carried out in HP-Z400-Workstation dual booting system where Linux - Ubuntu version with 16.04 LTS is used with the memory of 99.3 GB. The RAM of the system was 16 BGB with a processor of Intel^®^ Xeon(R) CPU W3565 at 3.20GHz × 4 having NVC1 graphics.

## Data availability statement

The original contributions presented in the study are publicly available. This data can be found here: Python library: PyPi (https://pypi.org/project/sielu/). Web-based application: http://login1.cabgrid.res.in:5500/ Mobile Application: download from http://login1.cabgrid.res.in:5500/.

## Author contributions

SJ, AR, and DK conceived the theme of the study. BA, MI, SJ, and AR developed the methodology, BA collected the data. BA, MH, SJ, MI, and UA were involved in the computational analysis and development of web resources and mobile applications. SJ, MI, and AR supervised the study. BA wrote the original draft. DK and AR reviewed and edited the manuscript. All authors contributed to the article and approved the submitted version.

## Supplementary material

Supplementary Figure 1 Precision-Recall curve of different abiotic stress data.

Supplementary Figure 2 Receiver Operating Characteristics curve of different abiotic stress data.
