# Supplementary figures and images for: DeepAProt: Deep learning based abiotic stress protein sequence classification and identification tool in cereals
Source: Front Plant Sci. 2023 Jan 12;13:1008756. doi: 10.3389/fpls.2022.1008756 (PMC9877618; doi:10.3389/fpls.2022.1008756)

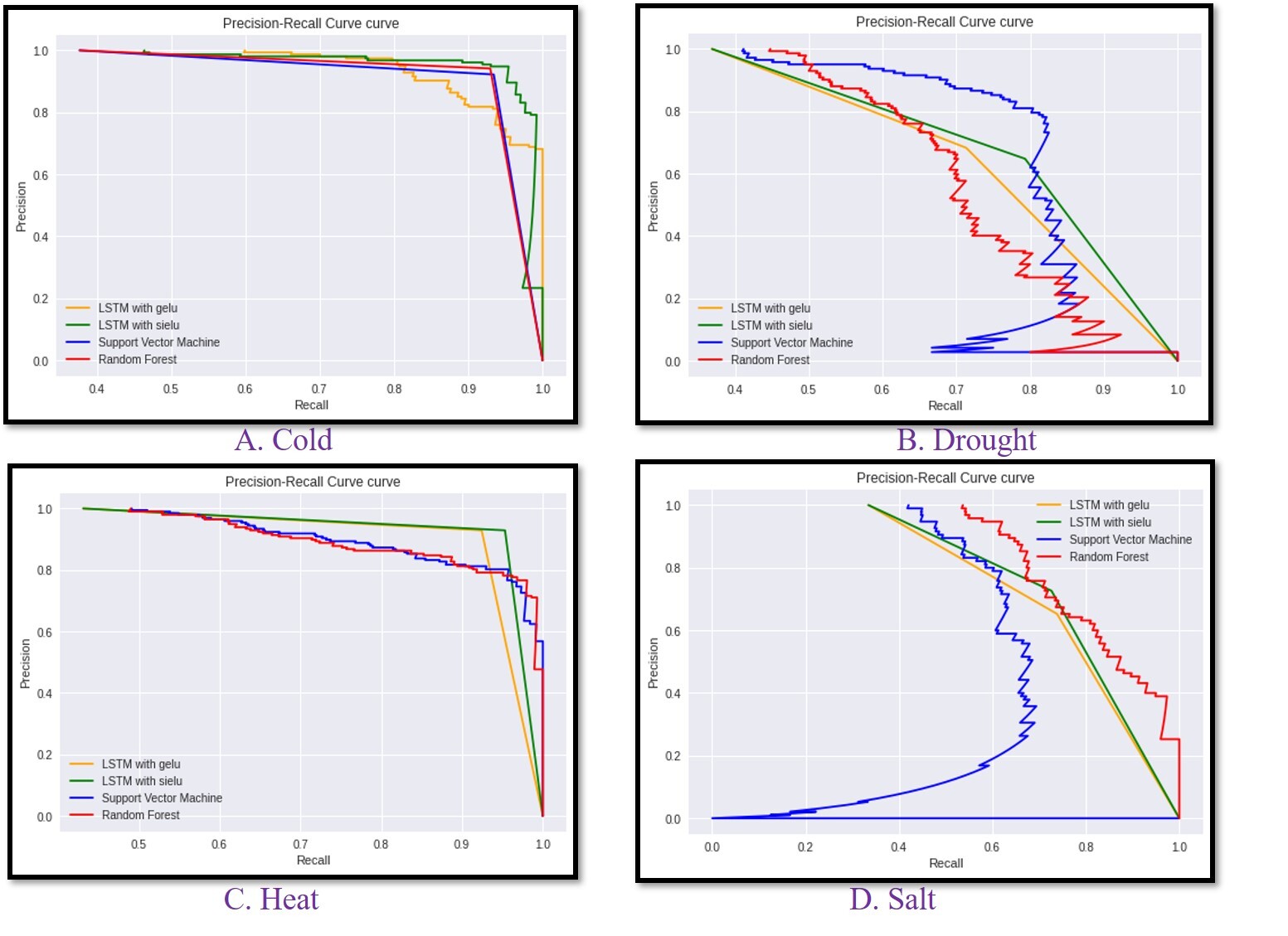

Supplement: Supplementary Figure 1 — Precision-Recall curve of different abiotic stress data. [file Image_1.jpg]

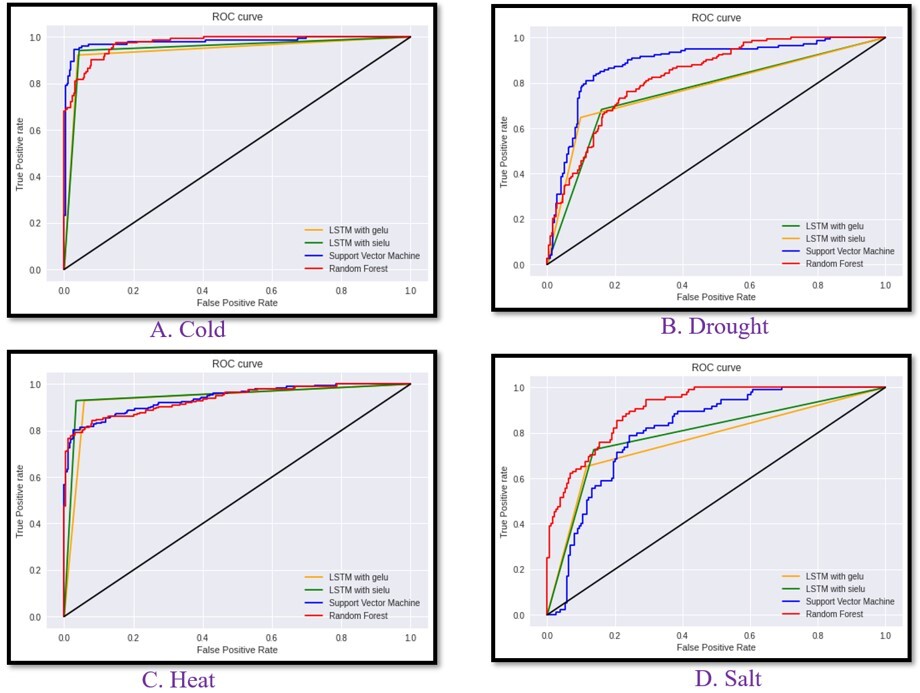

Supplement: Supplementary Figure 2 — Receiver Operating Characteristics curve of different abiotic stress data. [file Image_2.jpg]
